# Genome downsizing and karyotype constancy in diploid and polyploid congeners: a model of genome size variation

**DOI:** 10.1093/aobpla/plu029

**Published:** 2014-06-26

**Authors:** Lidia Poggio, María Florencia Realini, María Florencia Fourastié, Ana María García, Graciela Esther González

**Affiliations:** Instituto de Ecología, Genética y Evolución (IEGEBA)-Consejo Nacional de Investigaciones Científicas y Técnicas (CONICET) and Laboratorio de Citogenética y Evolución (LaCyE), Departamento de Ecología, Genética y Evolución, Facultad de Ciencias Exactas y Naturales, Universidad de Buenos Aires, Ciudad Autónoma de Buenos Aires, Argentina

**Keywords:** Bimodal karyotype, DNA amount variation, genome size, *Hippeastrum*, karyotype constancy, polyploids.

## Abstract

Evolutionary chromosome change involves significant variation in DNA amount in diploids and genome downsizing in polyploids. Genome size and karyotype parameters of *Hippeastrum* species with different ploidy level were analysed. In *Hippeastrum*, polyploid species show less DNA content per basic genome than diploid species. The rate of variation is lower at higher ploidy levels. All the species have a basic number *x* = 11 and bimodal karyotypes. The basic karyotypes consist of four short metacentric chromosomes and seven large chromosomes (submetacentric and subtelocentric). The bimodal karyotype is preserved maintaining the relative proportions of members of the haploid chromosome set, even in the presence of genome downsizing. The constancy of the karyotype is maintained because changes in DNA amount are proportional to the length of the whole-chromosome complement and vary independently in the long and short sets of chromosomes. This karyotype constancy in taxa of *Hippeastrum* with different genome size and ploidy level indicates that the distribution of extra DNA within the complement is not at random and suggests the presence of mechanisms selecting for constancy, or against changes, in karyotype morphology.

## Abstract (precis)

In several genus evolutionary chromosome change involves variation in DNA amount in diploids and genome downsizing in polyploids. The constancy of bimodal karyotypes, even with changes in ploidy level and DNA content per basic genome indicate that the distribution of DNA within the complement is not at random and suggest the presence of mechanisms selecting for constancy, or against changes, in the karyotype morphology.

## Introduction

The diversity of plant genomes is manifested through a wide range of chromosome number and genome size (Leitch and Leitch 2013). The partitioning of total DNA in chromosomes is a complex level of structural and functional organization of nuclear genomes. Each species has a characteristic chromosome complement, its karyotype, which represents the phenotypic appearance of somatic chromosomes. Karyotype features more commonly recorded for comparative evolutionary analysis are number and size of the chromosomes, position and type of primary and secondary constrictions, karyotype symmetry and genome size, among others. Genome size does not necessarily reflect chromosome number variation since mechanisms producing changes in total DNA amount are different for those leading to changes in chromosome number. The increases in genome size arise predominantly through polyploidy and amplification of non-coding repetitive DNA, especially retrotransposons (Bennetzen *et al.* 2005). These mechanisms are counterbalanced by processes that result in a decrease in genome size such as unequal recombination and illegitimate recombination (Leitch and Leitch 2013). Genome size changes (amplification or deletions) are correlated with karyotype parameters and can affect the entire chromosome complement or they may be restricted to a subset of chromosomes.

Different patterns of distribution of DNA among chromosomes or chromosome arms, even in the absence of chromosomal rearrangements, could lead to important changes in the karyotype parameters, mainly in the asymmetry (Peruzzi *et al.* 2009). This parameter refers to karyotypes with a predominance of chromosomes with terminal/subterminal centromeres (intrachromosomal asymmetry) and highly heterogeneous chromosome sizes (interchromosomal asymmetry) (reviewed by Peruzzi and Eroglu 2013). It is interesting to point out that evolutionary chromosome change involving alteration in DNA amount does not always lead to changes in the morphology of the karyotype, given that in several groups of plants karyotype orthoselection has been found (White 1973), as was described in Asparagaceae, Xanthorrhoeacae (Brandham 1971; Brandham and Doherty 1998) and *Vicia* (Fabaceae) (Naranjo *et al.* 1998), among others.

The bimodal karyotype represents a special case of asymmetry and is characterized by the presence of two sharply distinct classes of chromosomes without a gradual transition. The bimodal karyotype has been reported in monocots such as Xanthorrhoeacae (*Aloe*, *Haworthia*, *Gasteria*), Asparagaceae (*Agave*, *Yucca*) and Amaryllidaceae (*Hippeastrum*, *Rodophiala*) (Naranjo 1969; Brandham 1971; Naranjo and Andrada 1975; Arroyo 1982; Naranjo and Poggio 1988; Brandham and Doherty 1998; Vosa 2005; Poggio *et al.* 2007; Weiss-Schneeweiss and Schneeweiss 2013). Taxonomic groups with bimodal karyotypes and genome size variation offer the opportunity to analyse the nature and distribution of changes between chromosome arms and among members of the haploid chromosome set.

*Hippeastrum* Herb. is a genus of perennial and bulbous plants of the tribe Hippeastreae of Amaryllidaceae J.St.-Hil. (Meerow *et al.* 2000) with *ca.* 60 species inhabiting tropical and subtropical America from Mexico and the Antilles to central Argentina. Their species have economic value as ornamentals and are used in the pharmaceutical industry due to their high content of alkaloids. In the genus *Hippeastrum*, chromosomes of about 41 species have been studied and all presented bimodal karyotypes and a basic number *x* = 11. The karyotypes consist of four short metacentric (m) chromosomes and seven large chromosomes (four submetacentric—sm and three subtelocentric—st) (Naranjo 1969; Naranjo and Andrada 1975; Arroyo 1982; Brandham and Bhandol 1997). This genus is an interesting model to analyse how and where gain or loss of DNA occurs, and how these changes affect karyotype morphology.

Poggio *et al.* (2007) found, in 12 *Hippeastrum* diploid species from South America, karyotypes similar to that previously described but significant differences in nuclear DNA content. These authors report that karyotype constancy is a product of changes in DNA content occurring in the whole-chromosome complement, and that DNA addition to the long and short sets of chromosomes varies independently. The authors state that the evolutionary changes in DNA amount are proportional to chromosome length, maintaining karyotype uniformity. They found that in diploid species with higher DNA content, the short chromosomes add equal DNA amounts to both arms, maintaining their metacentric morphology, whereas the long chromosomes add DNA only to the short arm, increasing chromosome symmetry.

Several authors reported variation in ploidy level (3*x* to 7*x*) in several species of the genus (Sato 1938; Neto 1948; Naranjo 1969; Lakshmi 1980; Arroyo 1982; Beltrao and Guerra 1990; Zou and Quin 1994). It is interesting to point out that several polyploids previously analysed were considered to be autopolyploids, because they have similar basic bimodal karyotypes to those described in diploid species (Naranjo 1969; Naranjo and Andrada 1975). The genome size of the polyploid species of *Hippeastrum* has not yet been reported. It has been frequently documented that the major trend in vascular plants is a decrease in the genome size per haploid genome (1Cx), when a polyploidization event occurs (Leitch and Bennett 2004; Leitch and Leitch 2013). This genome downsizing, which could be involved in the genetic and cytogenetic diploidization of polyploids, consists in non-random deleting of coding and non-coding sequences, changes in retroelements, chromosome reorganization, gain or loss of chromosomes or entire genomes, altered patterns of gene expression and epigenetic modifications (Feldman and Levy 2005; Ma and Gustafson 2006; Jones and Langdom 2013; Leitch and Leitch 2013).

In the present work, variation of DNA amount in species of *Hippeastrum* with different ploidy level is presented with the aim to evaluate if genome size per haploid genome decreases when a polyploidization event occurs. Besides, karyotype parameters are evaluated to analyse if bimodality and karyotype' constancy detected in diploids can still take place in different ploidy levels, even in the presence of genome downsizing. Finally, the variation in DNA content and correlated karyotype parameters will be discussed in the different ploidy levels studied.

## Methods

Cytological studies were carried out on material cultivated at the Royal Botanic Gardens, Kew, with the exception of one specimen of *Hippeastrum argentinum* that was collected by A. T. Hunziker (ATH 18258). The sources of the materials are listed in Table 1.

**Table 1.** Origin, accession numbers and ploidy level of the *Hippeastrum* species.

Species	Ploidy level	Origin	Kew accession or Herba Nt.
*H. machupijchense* (Vargas) Hunt	2*x*	Perú, Cuzco, Machupichu	376-76-03600
*H. solandriflorum* Herb.	2*x*	Argentina, Corrientes	301-79-02627
*H. aulicum* Herb.	2*x*	Brazil, Santa Catarina	434-79-04428
*H. hybrid* Sealy	2*x*	Brazil	344-79-03154
*H. argentinum* (Pax) Hunz.	2*x*	Argentina, Catamarca	ATH18258
*H. psittacinum* (Ker Gawl.) Herb.	2*x*	Brazil	088-60-08801
*H. evansiae* (Traub & Nels.) Moore	2*x*	Bolivia	302-79-02858
*H. tucumanum* Holmb.	2*x*	Argentina, Tucumán	361-75-03430
*H. parodii* Hunz. & Coc.	2*x*	Argentina, Corrientes, Três Cerros	400-76-03888
*H. correiense* (Bury) Worsley	2*x*	Brazil, Sao Paulo	419-72-03854
*H. rutilum* (Ker Gawl.) Herb.	2*x*	Brazil	501-66-50111
*H. morelianum* (Lamaire) Traub	2*x*	Brazil, Sao Paulo, Serra do Mar	419-72-03853
*H. puniceum* (Lamb.) Kuntze	3*x*	Guyana, Mt Roraina, Kako	236-80-02247
*H. reginae* (L.) Herb.	4*x*	Peru, Cuzco, Marcapata	408-53-40803
*H. rutilum* (Ker Gawl.) Herb.	4*x*	Brazil	006-69-16919
*H. starkii* (Nels. & Traub) Moore	4*x*	Bolivia	487-67-48702
*H. blossfeldiae* (Traub & Doran) Vam Scheepen	4*x*	Brazil, Sao Paulo	139-74-01555
*H. scopulorum* Baker	5*x*	Bolivia, La Paz	037-72-00389
*H. rutilum* (Ker Gawl.) Herb.	5*x*	Brazil, Pelotas	396-70-03892
*H. cybister* (Herb.) Benth. ex Baker	5*x*	Brazil	418-72-09675
*H. puniceum* (Lamb.) Kuntze	6*x*	Brazil, Sao Paulo, Araras	277-78-030023

### Cytological analysis

For squashing, root tips were pretreated for 2.5 h in 0.002 M 8-hydroxyquinoline at 20 °C, fixed in 3 : 1 absolute ethanol : acetic acid and stained in Feulgen solution. The average of centromeric indices, for small and large chromosomes (CI_S_ and CI_L_), was calculated according to Poggio *et al.* (2007). The nomenclature used for chromosome morphology is that proposed by Levan *et al.* (1964). To estimate karyotype asymmetry, the coefficient of variation of chromosome length (CV_CL_) and the mean centromeric asymmetry (M_CA_) were calculated according to Peruzzi and Eroglu (2013). The A1 and A2 indices from Romero Zarco (1986) were also calculated for comparison with previously published data in *Hippeastrum* and related genera. Chromosomal parameters were measured using the freeware program MicroMeasure 3.3 (http://www.colostate.edu/Depts/Biology/MicroMeasure/). Mean values for the karyotype parameters were measured from a minimum of five scattered metaphase plates in each accession.

### Feulgen staining and cytophotometry

Root tips were fixed in 3 : 1 absolute ethanol : acetic acid for 1–4 days. The staining method was performed as described in Tito *et al.* (1991). The amount of Feulgen staining per nucleus, expressed in arbitrary units, was measured at a wavelength of 550 nm using the scanning method on a Vickers M85 Microspectrophotometer (Jodrell Laboratory, RBG, Kew, UK). The DNA content per basic genome expressed in picograms (pg) was calculated using *Allium cepa* var. *Ailsa Craig* as a standard (2C = 33, 55 pg; Bennett and Smith 1976). DNA content was measured in 25–50 telophase nuclei (2C) per accession.

### Statistical analysis

The differences between species in 1Cx DNA content were tested through an analysis of variance (ANOVA) using generalized linear mixed models. The mean values of genome sizes were calculated and multiple contrasts were performed with the LSD Fisher method (Fisher 1932). These statistical analyses were considered significant if their *P* values were <0.05.

The relationship between total DNA content and ploidy level was studied by fitting a weighted least-squares linear regression. This method compensates for the variable number of DNA measurements available for each species and ploidy level (Aitken 1935).

The statistical analyses were performed using the Infostat program, FCA, National University of Córdoba (Di Rienzo *et al.* 2012) and the R programming language (R Development Core Team 2004).

## Results

Total genome size (2C), DNA per basic genome (1Cx), karyotype formulae and karyotype parameters for diploid and polyploid species are listed in Table 2.

**Table 2.** Chromosome numbers, genome sizes and karyotype parameters of the *Hippeastrum* species. 2C DNA, total genomic DNA; 1Cx DNA, DNA per basic genome; CI_S_, average of centromeric index of short chromosomes; CI_L_, average of centromeric index of long chromosomes; A1, intrachromosomal asymmetry index; A2, interchromosomal asymmetry index; M_CA_, mean centromeric asymmetry; CV_CL_, coefficient of variation of chromosome length; CV_S_, volume of short chromosomes as a percentage of the volume of all chromosomes. Means with the same letter are not significantly different (*P* ≤ 0.05). *Data taken from Poggio *et al.* (2007), except for M_CA_ and CV_CL_ values.

Species	2*n*	2C DNA (pg) (X ± SE)	1Cx DNA (pg) (X ± SE)	CI_S_	CI_L_	A1	A2	M_CA_	CV_CL_	CV_S_ ( %)	Karyotype formula
H. machupijchense*	22	34.17 (±0.20)	17.08 (±0.10)^A^	42.42	26.17	0.50	0.30	–	30.56	23.65	[4m] + 4sm + 3st
H. solandriflorum*	22	33.77 (±0.50)	16.88 (±0.25)^AB^	42.48	24.39	0.51	0.31	36.00	31.03	23.59	[4m] + 4sm + 1sm–st + 2st
H. psittacinum*	22	31.34 (±0.23)	15.67 (±0.12)^E^	45.85	25.37	0.48	0.32	–	32.03	24.85	[4m] + 3sm + 1sm–st + 3st
H. evansiae*	22	30.92 (±0.28)	15.46 (±0.14)^EF^	46.87	23.83	0.47	0.32	36.08	32.20	23.24	[4m] + 3sm + 1sm–st + 2st + 1st–t
H. tucumanum*	22	30.64 (±0.17)	15.32 (±0.09)^FG^	43.20	24.89	0.50	0.31	39.24	31.01	24.90	[4m] + 3sm + 1sm–st + 3st
H. parodii*	22	30.21 (±0.23)	15.11 (±0.11)^G^	42.46	23.27	0.52	0.29	37.04	29.20	23.91	[4m] + 3sm + 1sm–st + 3st
H. correiense*	22	29.05 (±0.25)	14.53 (±0.13)^H^	45.58	22.78	0.51	0.29	35.46	29.04	24.44	[4m] + 2sm + 2sm–st + 1st + 2t
*H. rutilum*	22	27.98 (±0.28)	13.99 (±0.14)^I^	45.10	22.38	0.51	0.31	33.57	31.03	23.97	[4m] + 2sm + 1sm–st + 3st + 1t
H. morelianum*	22	26.80 (±0.19)	13.40 (±0.09)^J^	43.75	19.99	0.55	0.32	37.39	32.08	23.21	[4m] + 2sm + 1sm–st + 2st + 2t
*H. puniceum*	33	38.69 (±0.48)	12.90 (±0.16)^K^	44.76	23.97	0.49	0.30	31.88	30.33	24.14	[4m] + 1sm + 3sm–st + 2st + 1t
*H. reginae*	44	52.79 (±0.30)	13.20 (±0.08)^J^	–	–	–	–	–	–	–	–
*H. rutilum*	44	48.93 (±0.37)	12.23 (±0.09)^L^	42.63	23.23	0.54	0.32	39.75	32.02	23.14	[3m + 1m–sm] + 1sm + 2sm–st + 3st + 1t
*H. starkii*	44	47.19 (±0.30)	11.80 (±0.08)^M^	–	–	–	–	–	–	–	–
*H. blossfeldiae*	44	46.04 (±0.29)	11.51 (±0.07)^N^	42.85	23.18	0.53	0.32	39.30	32.01	23.05	[3m + 1m–sm] + 2sm + 1sm–st + 3st + 1t
*H. scopulorum*	55	58.71 (±0.26)	11.74 (±0.05)^M^	–	–	–	–	–	–	–	–
*H. rutilum*	55	58.20 (±0.42)	11.64 (±0.10)^MN^	45.26	24.37	0.49	0.29	35.62	29.02	24.69	[4m] + 3sm–st + 4st
*H. cybister*	55	56.35 (±0.38)	11.20 (±0.11)^O^	45.23	23.15	0.50	0.30	37.55	30.04	25.01	[4m] + 1sm + 3sm–st + 3st
*H. puniceum*	66	64.67 (±0.41)	10.78 (±0.07)^P^	44.88	34.10	0.42	0.33	28.61	33.01	25.12	4m + 3sm + 3 sm–st + 1 st

**Figure 1.**
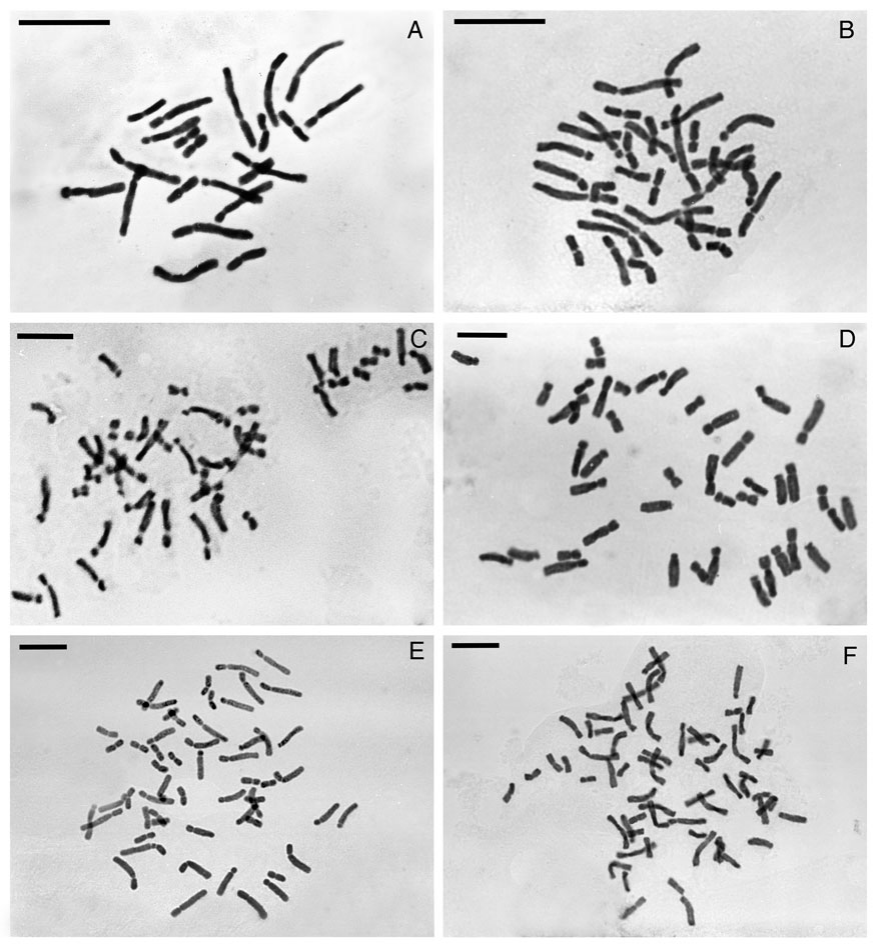
Mitotic metaphases of *Hippeastrum* species: (A) *H. rutilum* (2*n* = 22), (B) *H. puniceum* (2*n* = 33), (C) *H. rutilum* (2*n* = 44), (D) *H. blossfeldiae* (2*n* = 44), (E) *H. cybister* (2*n* = 55) and (F) *H. puniceum* (2*n* = 66). Scale bar: 10 µm.

All the diploid and polyploid species presented *x* = 11 (Table 2 and Fig. 1). The karyotype formulae and parameters show a basic bimodal karyotype, with the presence of two distinct classes of chromosomes, long and short (Figs 1 and 2). The relative chromosome sizes and relative arm sizes per basic haploid complement (*x* = 11) are given in a diagrammatic form (Fig. 2). The volume of the short chromosomes as a percentage of the volume of all chromosomes (CV_S_) is similar in all the taxa analysed (23.05–25.12) (Table 2). The centromeric indices of short chromosomes (CI_S_) are very similar among diploid and polyploid taxa (42.42–46.87). On the other hand, the centromeric indices of large chromosomes (IC_L_) decrease at lower genome size in diploids (19.9–26.17), while 3*x*, 4*x* and 5*x* present similar values (23.18–24.37). The hexaploid differs from the rest of the species in their karyotype parameters, having a similar CI_S_ but a higher CI_L_ (Table 2). The karyotype asymmetry indices M_CA_ and CV_CL_ are given in Table 2 and are plotted against DNA content in Fig. 3. In this figure, it can be seen that *Hippeastrum puniceum* (6*x*), with the lowest basic DNA amount (1Cx), occupies an isolated position when compared with the rest of the *Hippeastrum* species. This is a consequence of its more symmetrical karyotype.

**Figure 2.**
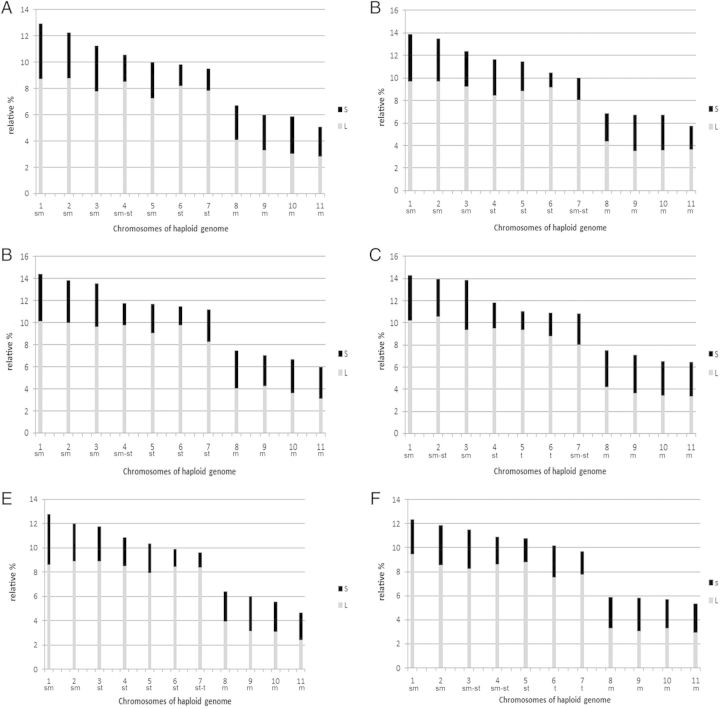
Relative chromosome and arm sizes per haploid complement (*x* = 11): (A) *H. solandriflorum* (2*x*), (B) *H. tucumanum* (2*x*), (C) *H. parodii* (2*x*), (D) *H. correiense* (2*x*), (E) *H. rutilum* (2*x*), (F) *H. morelianum* (2*x*), (G) *H. puniceum* (3*x*), (H) *H. rutilum* (4*x*), (I) *H. blossfeldiae* (4*x*), (J) *H. cybister* (5*x*), (K) *H. rutilum* (5*x*) and (L) *H. puniceum* (6*x*). S, short arm; L, long arm; m, metacentric; sm, submetacentric; st, subtelocentric; t, telocentric.

**Figure 2.**
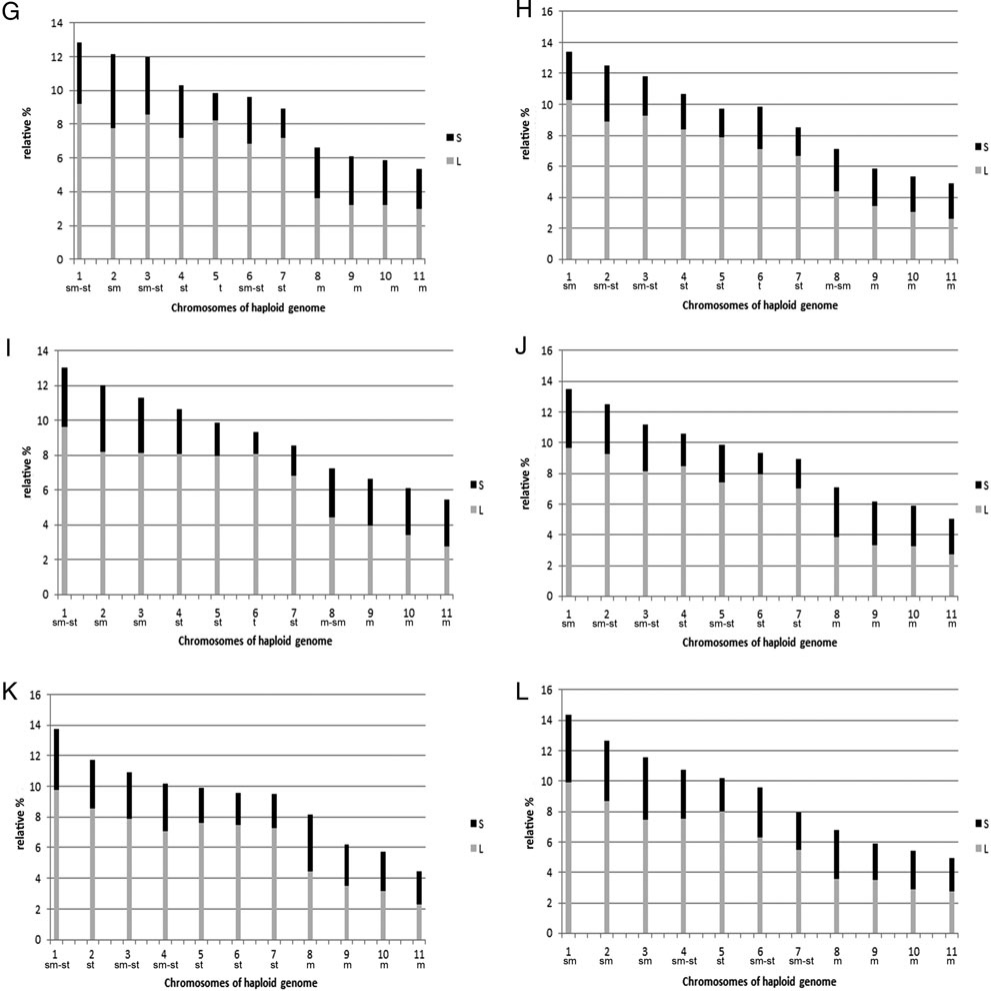
Continued.

**Figure 3.**
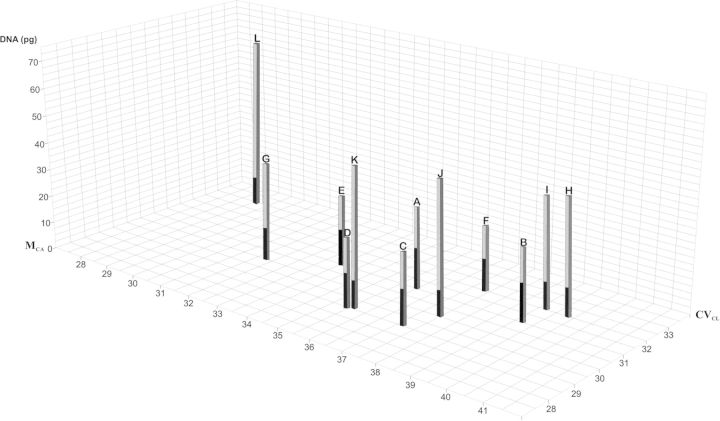
Asymmetry parameters (M_CA_ and CV_CL_) plotted against DNA content. The bars represent the total DNA amount (2C) and the black zone indicates the basic DNA amount (1Cx). (A) *H. solandriflorum* (2*x*), (B) *H. tucumanum* (2*x*), (C) *H. parodii* (2*x*), (D) *H. correiense* (2*x*), (E) *H. rutilum* (2*x*), (F) *H. morelianum* (2*x*), (G) *H. puniceum* (3*x*), (H) *H. rutilum* (4*x*), (I) *H. blossfeldiae* (4*x*), (J) *H. cybister* (5*x*), (K) *H. rutilum* (5*x*) and (L) *H. puniceum* (6*x*). M_CA_, mean centromeric asymmetry; CV_CL_, coefficient of variation of chromosome length.

**Figure 4.**
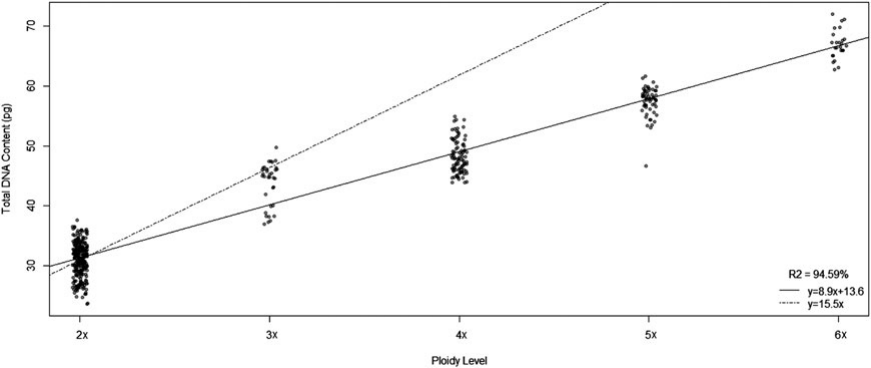
Total DNA content (2C) plotted against ploidy level. Solid line, linear fit; broken line, extrapolated from diploids.

**Figure 5.**
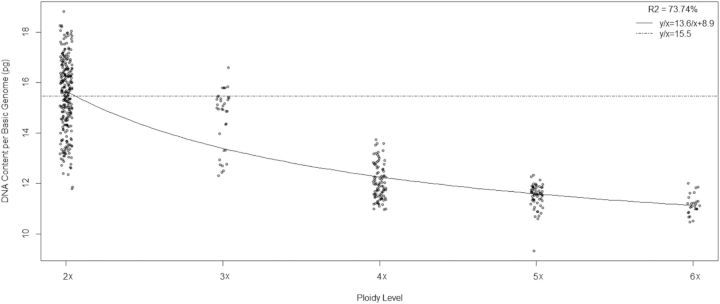
DNA content per basic genome (1Cx) plotted against ploidy level. Solid line, linear fit/ploidy level (*x*); broken line, extrapolated from diploids/ploidy level (*x*).

Significant differences in 1Cx DNA amount were found among the taxa (*F* = 427.44, *P* < 0.0001). They are indicated in Table 2. The total DNA content (2C) increases with ploidy level (DNA 2C: *y* = 8.9*x* + 13.6; *x* = ploidy level, *R*^2^ = 95 %) but the calculated regression line has a gentler slope than the line extrapolated from the diploid mean, which assumes that when the number of genomes increases DNA is added as an exact multiple of the DNA content per basic genome (Fig. 4). When DNA content per basic genome is plotted against ploidy level, a hyperbolic curve is obtained (1Cx: *y*/*x* = 13.6/*x* + 8.9) (Fig. 5). This new formula results from rearranging the linear regression equation of Fig. 4.

## Discussion

In the present work, genome size and karyotype parameters of *Hippeastrum* species with different ploidy level were analysed and compared with previous data.

Total DNA (2C) varies from 26.80 to 34.17 pg among diploids and increases with ploidy level, reaching a value of 64.67 pg in the hexaploid species. This genus has large genomes, since according to Leitch *et al.* (1998) most angiosperms actually have small 1C values (from 0.1 to 3.5 pg).

DNA per basic genome (1Cx), calculated from total DNA content, varies from 17.08 to 13.40 pg in diploids. The difference between these extreme values is significant. In polyploids there is a gradual decrease in the 1Cx value when ploidy level increases, varying from 12.90 pg in triploids to 10.78 pg in hexaploids. In *Hippeastrum* the polyploids studied show less DNA content per basic genome than diploids. Considering the average of basic DNA content for diploids, the triploid diminishes by 16.77 % while the decrease among 3*x*–4*x*, 4*x*–5*x* and 5*x*–6*x* ploidy levels is lower, varying between 5.5 and 6.5 %. These results show that in *Hippeastrum*, DNA per haploid genome decreases in polyploids, the rate of variation being lower at higher ploidy levels. Many examples are found in the literature where polyploidy is associated with decreasing genome size, in terms of DNA content per haploid genome. Moreover, comparative genome studies have shown that the downsizing of the genome can take place even in a few generations and could be involved in the genetic and cytogenetic diploidization (Soltis *et al.* 2003; Kellogg and Bennetzen 2004; Leitch and Bennett 2004; Feldman and Levy 2005; Ma and Gustafson 2006; Leitch and Leitch 2013). While polyploidy, joined with transposable element amplification, is widely considered to play a role in generating increased genome size, mechanisms that generate small deletions such as unequal homologous recombination and illegitimate recombination could be involved in genome downsizing (Bennetzen *et al.* 2005; Leitch and Leitch 2013). To explain this widespread phenomenon it could be postulated that at polyploid level, the DNA elimination leads to a more adequate balance between total DNA content and certain cellular parameters. Moreover, at polyploid level, the partial elimination of DNA sequences is more easily tolerated. However, in some cases, as in genus *Larrea* (Zygophyllaceae) (Poggio *et al.* 1989) or *Aloe* (Xanthorrhoeacae) (Brandham and Doherty 1998), differences in 1Cx at different ploidy levels are not statistically significant.

The diploid and polyploid species of *Hippeastrum* here studied presented *x* = 11 and despite possessing significant differences in their genome size, all have a basic bimodal karyotype with four small m and seven large sm/t chromosomes. The constancy of the karyotype in taxa of *Hippeastrum* with different genome size and ploidy level indicates that the distribution of extra DNA within the complement is not at random and suggests the presence of mechanisms selecting for constancy, or against changes, in karyotype morphology, processes named by White (1973) as karyotype constancy or karyotype orthoselection, respectively. Several studies have shown that karyotype orthoselection in diploid species with significant differences in genome sizes involves proportional changes in all chromosomes, preserving the morphology of the complement (Brandham and Doherty 1998; Naranjo *et al.* 1998). Chromosomal parameters such as centromeric indices and karyotype asymmetry provide some insights into how the additional DNA is distributed in the genome, between small and large chromosomes as well as between arms of individual chromosomes. In this work we use M_CA_ and CV_CL_ to estimate the intrachromosomal and interchromosomal asymmetries, respectively (Peruzzi and Eroglu 2013). Moreover, we also employ the A1 and A2 indices from Romero Zarco (1986) only for comparative purposes with previous work in the *Hippeastrum* species and related genera (Naranjo and Poggio 1988; Poggio *et al.* 2007).

Different patterns of addition of DNA amount in a chromosome complement were reviewed by Peruzzi *et al.* (2009). For ‘proportional increase’, the amount of DNA added to each chromosome arm is proportional to its length. This pattern does not result in a change in karyotype asymmetry when genome size changes. This pattern has been observed in several genera, including *Aloe* and *Gasteria* (Brandham and Doherty 1998). For ‘equal increase’, the same amount of DNA is added to each chromosome arm regardless of its size. This will result in an increase in the intrachromosomal karyotype symmetry. Examples of genera showing this pattern include *Vigna* (Parida *et al.* 1990) and *Papaver* (Srivastava and Lavania 1991). In many genera of Liliaceae, Peruzzi *et al.* (2009) found an ‘unequal increase’, i.e. the amount of DNA added varies between longer and shorter chromosome arms unequally.

In *Hippeastrum*, with two sets of chromosomes that differ in size and morphology, a different pattern was observed. In diploid species the evolutionary changes in DNA amount occur in the whole-chromosome complement and are proportional to chromosome length, maintaining karyotype uniformity (Poggio *et al.* 2007). These authors analysed separately the CI of short and long chromosomes and proposed a model of genome size change where the DNA increase or decrease to the long and short sets of chromosomes varies independently.

In the diploid and polyploid species analysed here, the volume of short chromosomes as a percentage of the volume of all chromosomes (CV_S_) is very similar, indicating that the volume of long and short chromosomes remains in a similar proportion among species. As previously discussed, this karyotype uniformity occurs if changes are proportional to the relative length of each chromosome arm (Brandham 1983; Naranjo *et al.* 1998; Poggio *et al.* 2007). In diploid and polyploid species the CI_S_ are similar and did not show any relationship with DNA amount, varying from 42.60 to 46.80. This could be explained if the short chromosomes add or lose equal DNA amounts to both arms, maintaining their metacentric morphology. Diploid species with lower DNA content have minor CI_L_ indices and have more asymmetric karyotypes, with a greater number of long chromosomes st or t, i.e. the changes in DNA amount in the long chromosomes affect mainly in their short arms. Among the triploids, tetraploids and pentaploids variation in CI_L_ was not detected, being similar to that of the diploid species with lower DNA content. This could be attributed to the lower downsizing at higher ploidy level.

In the hexaploid species analysed here, CV_S_ and the bimodality are maintained, and CI_S_ values are similar to those of the diploid and polyploid species. However, a different pattern of changes is observed in the long chromosomes of its karyotype. CI_L_ is greater than that of the other studied species, indicating that centromeres have a more median position. While the number of chromosomes sm–st, st and t varies from 3 to 7 from diploids to pentaploids, the hexaploids have just one st chromosome. Moreover, it is the only species with m–sm long chromosomes, i.e. the subset of long chromosomes is more symmetrical. This could be explained if there is a threshold for the distribution of changes in the larger chromosomes when the chromosome number is >55. This threshold could be related to nuclear organization at the chromosome level, arrangement of nuclear territories, interactions among genomes to sharing a nucleus and disturbances during cell division. Anyway, still very little is known about the mechanisms and sequences involved in genome downsizing in *Hippeastrum*.

Navrátilová *et al.* (2003) reported that amplification of retroelement sequences is likely to increase the size of all chromosomes within the karyotype in an approximately equal manner. In *Hippeastrum*, the absence of notorious C and DAPI bands (unpubl. res.), joined to the presence of conserved bimodal karyotypes, even with changes in ploidy level and 1Cx value, strongly suggests that DNA changes could occur by amplification or deletion of retroelement sequences, which are generally dispersed in the genome.

## Conclusion

In the genus *Hippeastrum*, evolutionary chromosome change involves variation in DNA amount in diploids and genome downsizing in polyploids. Besides, the bimodal karyotype is preserved maintaining the relative proportions of members of the haploid chromosome set by karyotype orthoselection. The presence of conserved karyotypes, even with changes in ploidy level and DNA content per basic genome, is strongly susceptible to an adaptive interpretation, suggesting the existence of mechanisms that select for constancy in karyotype morphology.

## Sources of Funding

Funding was provided by grants from the Consejo Nacional de Investigaciones Científicas y Técnicas (CONICET-PIP 00342), Universidad de Buenos Aires (UBACYT 20020100100859) and Agencia Nacional de Producción Científica y Tecnológica—SECyT (PICT 2010-1665).

## Contributions by the Authors

All authors contributed to the experimental design, data analysis and manuscript preparation.

## Conflicts of Interest Statement

None declared.
